# An exact and interpretable test for detecting aging behavior in reliability and survival data

**DOI:** 10.1371/journal.pone.0349009

**Published:** 2026-07-08

**Authors:** Ibrahim A. Nafisah, Mohamed Kayid

**Affiliations:** Department of Statistics and Operations Research, College of Science, King Saud University, Riyadh, Saudi Arabia; Roma Tre University: Universita degli Studi Roma Tre, ITALY

## Abstract

The identification of aging behavior in reliability and survival data is a central problem with important implications for risk assessment, system reliability, and decision-making in applied fields. The exponential model serves as the standard benchmark corresponding to the absence of aging, making the detection of departures from this model a key statistical task. This paper develops an exact nonparametric test for detecting increasing failure rate in average (IFRA) type aging in complete lifetime data. The test is constructed through a deviation functional derived from the defining properties of the IFRA class, providing a structurally meaningful and practically interpretable measure of departure from exponentiality. A main contribution is the derivation of an exact finite-sample representation of the test statistic under the exponential model using a normalized spacings framework, allowing accurate inference without reliance on asymptotic approximations. An asymptotic normal characterization is also established to support efficient large-sample implementation. To enhance practical applicability, a scale-invariant formulation is developed to remove dependence on unknown parameters. The performance of the proposed method is evaluated through extensive Monte Carlo simulations under a range of alternative models, including linear failure rate, Makeham, Gamma, and Weibull distributions. The results demonstrate consistently strong empirical power and competitive performance relative to existing procedures across different sample sizes. The practical utility of the method is further illustrated through applications to real-world datasets, where it provides coherent and interpretable assessments of aging behavior. Overall, the proposed approach offers a statistically rigorous and practically relevant tool for detecting aging patterns in reliability and survival data, combining exact finite-sample validity, asymptotic tractability, and strong empirical performance. In practical terms, the test helps determine whether a system or population shows signs of aging (increasing average risk over time) or behaves as if failures occur randomly at a constant rate.

## 1 Introduction

Aging behavior is a fundamental feature of reliability and survival data, with direct implications for system performance, risk assessment, maintenance planning, and decision making in engineering, biomedical, industrial, and environmental applications. In many real-world settings, failure mechanisms evolve over time as a result of cumulative effects such as wear, stress, degradation, biological progression, or environmental exposure. These mechanisms often produce lifetime distributions that depart from the memoryless exponential model and exhibit some form of positive aging. Accordingly, the development of statistically rigorous, interpretable, and practically reliable tools for detecting aging behavior remains an important problem in lifetime data analysis. Among the aging concepts studied in reliability theory, the class of increasing failure rate in average (IFRA) distributions is particularly important because it provides a flexible framework for describing gradual deterioration. A stronger notion is the increasing failure rate (IFR) property. If *X* is a nonnegative lifetime random variable with cumulative distribution function *F*(*x*), survival function $\bar{F}(x)=1-F(x)$, and probability density function *f*(*x*), then the failure rate function is


$$\begin{array}{c} \lambda (x)=\frac{f(x)}{\bar{F}(x)},\;x>0 \end{array}$$

The distribution is said to be IFR if λ(x) is non-decreasing in *x*. The IFRA class is broader than the IFR class: every IFR distribution is IFRA, but the converse is not necessarily true. Thus, IFRA provides a more flexible description of aging, especially in applications where the instantaneous failure rate may fluctuate while the average tendency toward failure increases with time.

The exponential distribution provides the standard benchmark for the absence of aging. Under an exponential model, the failure risk remains constant over time, meaning that the future lifetime behavior does not depend on the current age of the item or subject. In contrast, an IFRA distribution describes a situation in which the average failure risk increases with age. In practical terms, an exponential model may represent failures driven mainly by random shocks, whereas an IFRA model may represent progressive deterioration, such as a mechanical component that gradually wears out or a biological condition whose risk increases over time. Therefore, testing whether observed lifetime data follow an exponential distribution or exhibit IFRA-type aging is a central task in reliability and survival analysis. Formally, the distribution of X is said to be IFRA if


$$\begin{array}{c} \bar{F}(bx)\geq [\bar{F}(x){]}^{b},\;x>0,\;0<b<1, \end{array}$$

or, equivalently, if the average failure rate Λ(x)/x is increasing in *x* > 0, where


$$\begin{array}{c} \Lambda (x)=-\log\bar{F}(x) \end{array}$$

denotes the cumulative hazard function. Equality corresponds to the exponential case; see Barlow and Proschan [1]. This characterization shows why IFRA is useful in applications: it preserves the essential idea of positive aging while allowing greater flexibility than the stricter IFR condition. From both methodological and applied perspectives, this makes testing exponentiality against IFRA alternatives a natural and important inferential problem. In this paper, we consider the hypothesis testing problem


$$\begin{array}{c} H_{0}:F(x)=1-e^{-\theta x},\;x>0,\;\theta >0, \end{array}$$

against the alternative that F belongs to the IFRA class but is not exponential. Under the null hypothesis, the data follow the no-aging exponential model. Under the alternative, the data exhibit IFRA-type aging, meaning that the average failure rate increases with time. Rejection of the null hypothesis therefore provides statistical evidence of progressive deterioration or increasing average risk.

The problem of testing exponentiality against positive aging alternatives has been extensively studied in the statistical literature. Early foundational work by Proschan and Pyke [2] established important results for testing monotone failure rate properties. Hollander and Proschan [3] introduced the total time on test (TTT) procedure, which remains one of the most widely used graphical and inferential tools for detecting aging behavior. Deshpande [4] proposed nonparametric tests for exponentiality against IFRA alternatives based on weighted empirical functionals, while Aly and Lu [5] developed a unified asymptotic theory for testing exponentiality against several aging classes, including NBU, IFR, and IFRA alternatives. Ahmad [6,7] introduced moment-based procedures related to aging families of distributions, and more recently, Alqefari [8] proposed entropy-based tests for IFRA alternatives. These contributions demonstrate the depth of the literature and the variety of statistical approaches developed for detecting aging behavior.

Beyond classical testing procedures, recent work in reliability engineering has increasingly emphasized data-driven metrics. Modern complex systems rely heavily on degradation modeling, condition monitoring, and intelligent maintenance planning. For instance, advanced frameworks have been developed to automate anomaly detection and fault tracking. A prominent example includes utilizing unmanned aerial vehicles (UAVs) to detect defects and diagnose faults in wind turbine blades [9]. Similarly, sensor health monitoring is critical in high-stakes environments like nuclear power plants. In these facilities, principal component analysis has been combined with improved deep neural networks to achieve accurate sensor anomaly detection and reconstruction [10]. Modern artificial intelligence has also been integrated to optimize complex operational ecosystems. Scholars have deployed distributed hierarchical reinforcement learning frameworks to address airline fleet maintenance scheduling [11]. This approach effectively resolves highly dynamic, large-scale scheduling issues. In addition, predicting structural degradation requires robust tracking over time. Hybrid deep learning architectures have successfully enhanced predictive accuracy for the remaining useful life of mechanical bearings operating under variable conditions [12]. These contemporary studies highlight the practical importance of tracking failure behaviors across diverse engineering fields. However, these methods typically leverage complex algorithmic and machine-learning frameworks for component-specific diagnostics. As a result, they operate without exact finite-sample statistical guarantees for broader underlying aging classes. The present paper fills this methodological gap in the reliability landscape. Rather than focusing on a localized system architecture, we establish a baseline, mathematically exact nonparametric test. This test is designed explicitly to verify IFRA-type aging behavior across complete lifetime datasets.

Despite the substantial literature on exponentiality testing, several methodological limitations remain. Many existing procedures are based on rank statistics, moment inequalities, or information-theoretic measures whose connection to the defining structure of the IFRA class is indirect. Consequently, the mechanism by which departures from exponentiality are detected may not always have a clear interpretation in terms of average failure rate monotonicity. In addition, several available tests rely mainly on asymptotic approximations, which may be less reliable in small or moderate samples. This is a particularly important issue in practical reliability and survival studies, where sample sizes are often limited. These limitations motivate the need for a test that is directly connected to the IFRA definition, interpretable in terms of aging behavior, and capable of providing accurate finite-sample inference under the exponential null hypothesis.

Motivated by these considerations, the present study develops an exact and interpretable functional test for exponentiality against IFRA alternatives. The proposed procedure is constructed from a deviation functional derived directly from the characterizing inequality of the IFRA class. This construction yields a structurally grounded measure of departure from the no-aging exponential benchmark and provides a clear interpretation in terms of average aging behavior.

The present study is structured around four central research questions: (i) whether a test for exponentiality against IFRA-type aging can be constructed directly from the defining structure of the IFRA class, rather than from an indirect rank-, moment-, or information-based measure; (ii) whether the null distribution of the resulting statistic can be obtained exactly in finite-samples through a normalized-spacings representation, thereby reducing dependence on large-sample approximations; (iii) whether the proposed procedure can be formulated in a scale-invariant manner so that it remains applicable when the exponential parameter is unknown; and (iv) how the test performs, relative to established rank-based, moment-based, and entropy-based competitors, in terms of empirical size control, power, and practical interpretability.

The proposed method contributes to the literature in several ways. First, it introduces a deviation functional explicitly linked to the monotonicity of the average failure rate, providing a transparent and interpretable measure of IFRA-type departure from exponentiality. Second, it derives an exact finite-sample representation of the test statistic under the exponential null hypothesis using normalized spacings. This representation enables accurate inference in small and moderate samples and distinguishes the proposed procedure from tests that rely primarily on asymptotic approximations. Third, the statistic has an L-statistic structure, which supports asymptotic normality and efficient large-sample implementation. Fourth, a scale-invariant formulation is developed to remove dependence on the unknown rate parameter. Finally, simulation results and real-data applications show that the proposed test maintains good empirical size control and achieves competitive or superior power across several standard reliability and survival models.

To summarize the core focus of this work, the main action of this study is to develop a mathematically exact and physically interpretable functional test for exponentiality against IFRA alternatives. The methodology explicitly deviates from previous strategies in two profound ways. First, from a structural perspective, classical methods assess departures from exponentiality via proxy variables whose relationship with the average failure rate is indirect. In contrast, our proposed test statistic measures deviations explicitly along the monotonicity curve of the average failure rate itself. Second, from an inferential perspective, existing tests frequently depend on asymptotic normality rules which degrade in accuracy when sample sizes are small. The present study surmounts this by deriving an exact finite-sample representation under the null model using independent exponential normalized spacings, guaranteeing robust and precise null calibration across all sample thresholds.

To clearly delineate the novelty of the proposed methodology, it is instructive to contrast its framework with existing rank-based, moment-based, and entropy-based tests commonly found in the reliability literature.

- Contrast with Rank-Based Tests: Classical rank-based procedures assess departures from exponentiality by looking exclusively at the relative ordering or positions of the data points. While distribution-free, they discard the exact magnitudes of individual survival lifetimes. Our test utilizes a normalized spacings framework that preserves the full metric scale of the observations, translating directly into superior statistical power against specific IFRA alternatives.
- Contrast with Moment-Based Tests: Moment-based tests rely on weighted inequalities of higher-order expectations to detect aging. Because these metrics average out data behavior across the entire distribution spectrum, they often fail to capture localized variations in the failure rate curve. In contrast, our test is constructed directly from the defining functional inequality of the IFRA class, rendering it highly sensitive to the structural progression of average failure rate monotonicity.
- Contrast with Entropy-Based Tests: Information-theoretic or entropy-based tests measure overall statistical uncertainty or informational distance (such as Kullback-Leibler or Tsallis deviations) from an exponential baseline. While mathematically elegant, entropic functionals do not possess a direct physical or structural interpretation tied to aging. Our test statistic bridges this gap by directly calculating physical deviations along the average failure rate curve, providing clear engineering interpretability alongside its exact finite-sample null representation.

The proposed test is developed under the classical assumption of independent and identically distributed lifetimes, which is standard in nonparametric testing for exponentiality and aging properties. We recognize, however, that some applied reliability and survival data may exhibit temporal dependence. Extensions to strictly stationary or weakly dependent lifetime sequences would require additional theoretical development, such as suitable mixing conditions, modified limiting arguments, or resampling procedures. Such extensions are therefore identified as an important direction for future research rather than assumed as part of the present framework.

The remainder of the paper is organized as follows. Section 2 introduces the proposed functional test statistic and discusses its main properties. Section 3 establishes the exact finite-sample representation under the exponential null hypothesis and derives the asymptotic distribution. Section 4 presents a Monte Carlo simulation study evaluating empirical size and power relative to competing tests. Section 5 illustrates the practical applicability of the proposed method using real-world datasets from reliability, survival, environmental, and geological contexts. Section 6 concludes the paper and outlines possible directions for future research.

## 2 An exact functional test for the IFRA property

In this section, we develop a rigorous functional framework for constructing an exact test of exponentiality against IFRA alternatives. The proposed methodology is based on a deviation functional that directly reflects the defining structural property of the IFRA class, thereby providing a transparent and theoretically grounded measure of departure from exponentiality. Unlike procedures based on purely empirical or indirect constructions, the present approach is intrinsically linked to the monotonicity of the average failure rate and thus admits a clear aging-based interpretation. Let


$$\begin{array}{c} ℰ=\left\{F(x)=1-e^{-\theta x},x\geq 0,\theta >0\right\} \end{array}$$

denote the class of exponential distributions. As stated earlier, our goal is to test


$$\begin{array}{c} H_{0}:F\in ℰ\text{ \textasciitilde{}versus\textasciitilde{} }H_{1}:F\in IFRA\setminus ℰ \end{array}$$

based on an independent and identically distributed sample $X_{1},X_{2},\ldots ,X_{n}$ drawn from an absolutely continuous distribution function *F* with corresponding density *f*. Thus, under the null hypothesis the underlying lifetime distribution is exponential, whereas under the alternative it belongs to the IFRA class but is not exponential. Throughout this section, we maintain the classical independent and identically distributed (i.i.d.) assumption. Nevertheless, in some applications, lifetime observations may exhibit temporal dependence. In such cases, extensions to strictly stationary or weakly dependent observations may be possible under additional assumptions, such as suitable mixing conditions or resampling-based calibration. However, such extensions require separate theoretical development. In the present paper, the exact finite-sample representation is established under the classical i.i.d. framework, which is essential for the normalized-spacings argument used below.

To construct the test statistic, we introduce a deviation functional that quantifies departures from the exponential benchmark in the direction of IFRA aging. Define the cumulative hazard function as $\Lambda (x)=-log\overset{―}{F}(x).\;$The proposed functional is then defined by


$$\begin{array}{c} \begin{array}{c} T(F)=\int_{0}^{\infty }\;\;\int_{0}^{t}\;\;\left(\frac{\Lambda (t)}{t}-\frac{\Lambda (s)}{s}\right)stf(t)f(s)dsdt. \end{array} \end{array}$$ (2.1)

The functional *T*(*F*) is specifically designed to detect deviations from the constancy of the average failure rate Λ(x)/x. Under the null hypothesis, Λ(x)/x is constant and therefore *T*(*F*) = 0. Under strict IFRA alternatives, Λ(x)/x is increasing, implying that *T*(*F*)> 0. Hence, *T*(*F*) serves as a natural and theoretically justified measure of departure from exponentiality in the direction of IFRA aging. To obtain a more tractable representation, we expand the integrand in (2.1) to obtain


$$\begin{array}{c} T(F)=\int_{0}^{\infty }\;\Lambda (t)f(t)\int_{0}^{t}\;sf(s)dsdt-\int_{0}^{\infty }\;tf(t)\int_{0}^{t}\;f(s)\Lambda (s)dsdt. \end{array}$$

Rearranging the order of integration yields


$$\begin{array}{c} \begin{array}{c} T(F)=\int_{0}^{\infty }\;\;sf(s)\int_{s}^{\infty }\;\;f(t)\Lambda (t)dtds-\int_{0}^{\infty }\;\;tf(t)\int_{0}^{t}\;\;f(s)\Lambda (s)dsdt=I_{1}-I_{2}. \end{array} \end{array}$$ (2.2)

Using integration by parts, one may verify that


$$\begin{array}{c} \int_{s}^{\infty }\;f(t)\Lambda (t)dt=\bar{F}(s)+\bar{F}(s)\Lambda (s),\;s>0, \end{array}$$

and


$$\begin{array}{c} \int_{0}^{t}\;f(s)\Lambda (s)ds=F(t)-\bar{F}(t)\Lambda (t),\;t>0. \end{array}$$

Substituting these expressions, the functional reduces to


$$\begin{array}{c} \begin{array}{c} T(F)=\int_{0}^{\infty }\;\;tf(t)\left[2\bar{F}(t)\Lambda (t)+1-2F(t)\right]dt. \end{array} \end{array}$$ (2.3)

This representation clearly reveals the interaction between the survival and hazard components of the model. Under *H*_0_, the functional vanishes, whereas under IFRA alternatives it becomes strictly positive, thereby providing a structurally interpretable measure of deviation.

An important feature of the proposed functional is that it admits the L-functional representation


$$\begin{array}{c} \begin{array}{c} T(F)=\int_{0}^{\infty }\;\;tJ\left(F(t)\right)dF(t), \end{array} \end{array}$$ (2.4)

where the weight function J:(0,1)→ℝ is defined by


$$\begin{array}{c} \begin{array}{c} J(u)=-2(1-u)\log(1-u)+1-2u,\;0<u<1. \end{array} \end{array}$$ (2.5)

This representation shows that *T*(*F*) is a weighted functional of the distribution, where the function *J*(*F*(*t*)) captures the local contribution of each quantile to the overall deviation from exponentiality. Notably, J(u) is nonnegative on (0,1), further supporting the suitability of the proposed functional for detecting IFRA-type departures. For practical implementation, we define the empirical counterpart of *T*(*F*). Let $X_{\left(1\right)}<X_{\left(2\right)}<\cdots <X_{\left(n\right)}\;$denote the order statistics and let $F_{n}$ be the empirical distribution function. Substituting $F_{n}$ into (2.4), we obtain


$$\begin{array}{c} \begin{array}{c} \hat{T}=\int_{0}^{\infty }\;\;xJ\left(F_{n}(x)\right)dF_{n}(x)=\frac{1}{n}\sum_{i=1}^{n}\;\;J\left(\frac{i}{n}\right)X_{\left(i\right)}. \end{array} \end{array}$$ (2.6)

Thus, $\hat{T}$ is an L-statistic, inheriting desirable robustness and asymptotic properties while remaining computationally efficient. Since *T*(*F*) is not invariant under scale transformations, we normalize it by the mean μ=𝔼[X] and define the scale-free functional


$$\begin{array}{c} \begin{array}{c} T^{*}(F)=\frac{T(F)}{\mu }. \end{array} \end{array}$$ (2.7)

In practice, μ is replaced by the sample mean $\bar{X}$, leading to the scale-invariant test statistic


$$\begin{array}{c} \begin{array}{c} {\hat{T}}^{*}=\frac{\hat{T}}{\bar{X}}. \end{array} \end{array}$$ (2.8)

The following result establishes the fundamental positivity property of the proposed functional.

**Theorem 2.1.** If *F* is absolutely continuous and strictly IFRA, then *T*(*F*) > 0. Consequently, $T^{*}(F)$> 0.

**Proof.** For 0 < *s* < *t*, the s*t*rict IFRA property implies that Λ(t)/t> Λ(s)/s. Since *stf*(*t*)*f*(*s*) > 0, the integrand in (2.1) is strictly positive on a set of positive measure. Therefore, *T*(*F*) > 0. The positivity of $T^{*}(F)$ follows immediately.◻

Under IFRA alternatives, ${\hat{T}}^{*}$ is expected to take positive and increasingly large values. The null hypothesis is therefore rejected for sufficiently large values of ${\hat{T}}^{*}$, with critical values determined from its exact finite-sample null distribution, which will be developed in the next section.

### 2.1 Numerical illustration

To illustrate the direct computation of the proposed statistic, consider the ordered lifetime sample 4, 5, 6, 7, 8. For this sample, the sample mean is $\overset{―}{X}=6$. Using the empirical L-statistic representation in (2.6) with the weight function *J*(*u*), the computed value of the proposed statistic is $T_{n}=0.6795$. Hence, the corresponding scale-invariant statistic is $T_{n}^{*}=0.6795/6=0.1133$. This value is then compared with the critical value obtained under the exponential null distribution. This simple illustration shows that the statistic can be computed directly from the ordered observations and the corresponding weights, without fitting a parametric lifetime model.

## 3 Exact and asymptotic distributions of the test statistic

In this section, we establish the exact finite-sample distribution of the proposed test statistic under the null hypothesis of exponentiality and derive its asymptotic behavior by rewriting it as a linear combination of normalized spacings, thereby adopting the L-statistic approach originally developed by Langenberg and Srinivasan [13] and Box [14] for goodness-of-fit testing in the presence of aging alternatives. A key strength of the proposed methodology is that it admits an explicit probabilistic representation under the null model, enabling exact inference and sharply distinguishing it from existing procedures that rely primarily on asymptotic approximations. This dual capability, combining exact finite-sample characterization with asymptotic tractability, constitutes a central contribution of the present work.

### 3.1 Exact distribution under the null hypothesis

We begin by deriving the exact distribution of the statistic under *H*_0_. Let $X_{1},X_{2},\ldots ,X_{n}$ be a random sample from an exponential distribution with rate parameter θ>0, i.e.,


$$\begin{array}{c} F(x)=1-exp(-\theta x),\;x>0. \end{array}$$

A key structural property of exponential samples is that their normalized spacings are independent and identically distributed. Let $X_{\left(1\right)}<X_{\left(2\right)}<\cdots <X_{\left(n\right)}\;$denote the order statistics, and define the normalized spacings by


$$\begin{array}{c} D_{i}=(n-i+1)\left(X_{\left(i\right)}-X_{\left(i-1\right)}\right),\;i=1,2,\ldots ,n\text{ with }X_{\left(0\right)}=0. \end{array}$$

It is well established that the normalized spacings constitute a sequence of i.i.d. random variables, namely $D_{1},D_{2},\ldots ,D_{n}\sim_{i.i.d.}Exp(\theta ).\;$ This normalized-spacings representation provides a fundamental bridge between order statistics and independent exponential variables, and it is essential for deriving the exact finite-sample null distribution of the proposed statistic. Using this decomposition, the order statistics can be expressed as


$$\begin{array}{c} X_{\left(i\right)}=\sum_{j=1}^{i}\;\frac{D_{j}}{n-j+1},\;i=1,2,\ldots ,n. \end{array}$$

Substituting this representation into the definition of the statistic $\hat{T}$ in (2.6), we obtain


$$\begin{array}{c} \hat{T}=\frac{1}{n}\sum_{i=1}^{n}\;J\left(\frac{i}{n}\right)\sum_{j=1}^{i}\;\frac{D_{j}}{n-j+1}. \end{array}$$

Rearranging the order of summation yields the exact representation


$$\begin{array}{c} \begin{array}{c} \hat{T}=\sum_{j=1}^{n}\;\;a_{j}D_{j}, \end{array} \end{array}$$ (3.1)

where


$$\begin{array}{c} a_{j}=\frac{1}{n(n-j+1)}\sum_{i=j}^{n}\;\;J\left(\frac{i}{n}\right),\;j=1,2,\ldots ,n. \end{array}$$

This representation is of central importance: it shows that the proposed statistic can be written exactly as a linear combination of independent exponential random variables under the null hypothesis. This explicit representation provides a finite-sample basis for calibration that is not available for many existing tests against aging alternatives. Since the variables $D_{j}$ are independent and exponentially distributed, $\hat{T}$ follows a generalized hypoexponential distribution. Its moment generating function is given by


$$\begin{array}{c} \begin{array}{c} M_{\hat{T}}(t)=\prod_{j=1}^{n}\;\;\frac{\theta }{\theta -a_{j}t},\;t<\min_{1\leq j\leq n}\;\frac{\theta }{a_{j}}. \end{array} \end{array}$$ (3.2)

This explicit form provides a rigorous and computationally tractable basis for exact inference. In particular, critical values and *p*-values can be computed either analytically (when feasible) or through efficient Monte Carlo simulation without relying on asymptotic approximations. This is especially advantageous for small and moderate sample sizes, where asymptotic methods may perform poorly.

### 3.2 Scale-invariant formulation

To eliminate dependence on the unknown exponential parameter θ, we introduce the normalized statistic ${\hat{T}}^{*}=\hat{T}/\bar{X}$, where $\bar{X}=(1/n)\sum_{i=1}^{n}\;X_{i}$ is the sample mean. Under the null hypothesis, $\bar{X}$ is a consistent estimator of 1/θ, and therefore the ratio ${\hat{T}}^{*}$ is invariant with respect to the unknown exponential parameter.

### 3.3 Asymptotic distribution

We now establish the asymptotic behavior of the proposed statistic. To begin, we revisit a key result concerning the limiting behavior of linear functionals of the empirical distribution function.

**Theorem 3.2.** Consider a non-negative continuous random variable *X* with finite second moment $𝔼\left[X^{2}\right]<\infty$ and finite asymptotic variance $\sigma^{2}(J,F)<\infty$. Under these conditions, the following convergence in distribution holds as the sample size *n* grows:


n(T^−μ(J,F))σ(J,F)→d N(0,1) (3.3)

where


$$\begin{array}{c} \mu (J,F)=\int_{0}^{\infty }\;xJ\left(F(x)\right)dF(x), \end{array}$$

representing the population counterpart of the linear statistic. The asymptotic variance $\sigma^{2}(J,F)$ is given by


$$\begin{array}{c} \sigma^{2}(J,F)=\int_{0}^{\infty }\;\int_{0}^{\infty }\;J\left(F(x)\right)J\left(F(y)\right)\left[F\left(\min\left\{x,y\right\}\right)-F(x)F(y)\right]dxdy, \end{array}$$

with the score function J(·) as introduced in equation (6).

The given result in Theorem 3.2 follows immediately from Theorems 2 and 3 of Stigler [15], which furnish general sufficient conditions under which L-statistics, understood as linear functionals of the empirical distribution or linear combinations of order statistics, exhibit asymptotic normality. By invoking Slutsky s theorem together with Theorem 3.2, we obtain the limiting distribution of the scale-adjusted statistic ${\hat{T}}^{⋆}$.

**Theorem 3.3.** Assume the conditions of Theorem 3.2 hold. Then,


n(T^⋆−T(F)μ)→d N(0,σ2(J,F)μ2).

We now focus on the exponential null hypothesis. Because ${\hat{T}}^{⋆}$ is scale-invariant, its limiting distribution under *H*_0_ does not depend on the rate parameter. Consequently, we may set the rate to unity without loss of generality, taking $F(x)=1-e^{-x}$ for *x* > 0 (the standard exponential). Under this specification, we have *T*(*F*) = 0 and μ=1, leading to


$$\begin{array}{c} \sqrt{n}{\hat{T}}^{⋆}\to N(0,\sigma^{2}\;(J,F)). \end{array}$$ (3.4)

Interpretation of the test proceeds as follows: for sufficiently large *n*, markedly positive values of ${\hat{T}}^{⋆}$ signal increasing failure rate behavior (positive aging). Values near zero are compatible with exponentiality, while positive values would point toward increasing failure rate in average behavior. Since the asymptotic variance $\sigma^{2}(J,F)$ involves the unknown underlying distribution *F*, a consistent estimator must be substituted in practice. Following the approach of Jones and Zitikis [16], we adopt the empirical variance estimator


$$\begin{array}{c} {\hat{\sigma }}^{2}(J,F)=\sum_{i=1}^{n-1}\;\sum_{j=1}^{n-1}\;\pi (i,j)J\left(\frac{i}{n}\right)J\left(\frac{j}{n}\right)\delta_{i}\delta_{j}, \end{array}$$

where $\pi (i,j)=\left(\min\left(\frac{i}{n},\frac{j}{n}\right)-\frac{i}{n}\frac{j}{n}\right)$ and $\delta_{i}=X_{\left(i+1\right)}-X_{\left(i\right)}$ which retains consistency for $\sigma^{2}(J,F)$ under mild smoothness conditions on *J* and *F*. The resulting large-sample decision rule rejects *H*_0_ in favor of the IFRA alternative *H*_1_ at nominal level α whenever


$$\begin{array}{c} \frac{\sqrt{n}{\hat{T}}^{⋆}}{\hat{\sigma }(J,F)}>z_{1-\alpha }, \end{array}$$

where $z_{1-\alpha }$ denotes the upper (1−α) quantile of the standard normal distribution.

**Remark 3.2.** The present paper focuses on testing exponentiality against IFRA-type aging alternatives. Therefore, the rejection region is taken in the upper tail of the proposed statistic, where large positive values indicate increasing average failure risk.

These results justify the use of normal approximations for large samples and provide a theoretical basis for constructing asymptotic critical regions when exact computation is not required. The proposed test exhibits a distinctive and highly appealing combination of theoretical properties. It admits an exact finite-sample representation under the null hypothesis via a normalized spacings decomposition, enabling precise and reliable inference without reliance on asymptotic approximations. Moreover, its *L*-statistic structure guarantees asymptotic normality and supports accurate large-sample analysis. This unified theoretical foundation enhances the interpretability and practical applicability of the proposed methodology and distinguishes it from procedures that rely mainly on asymptotic calibration.

## 4 Simulation study

In this section, we investigate the finite-sample performance of the proposed statistic ${\hat{T}}^{*}$ through an extensive Monte Carlo simulation study. The objective is twofold: to evaluate the empirical power of the proposed test under a broad class of IFRA alternatives, and to benchmark its performance against several well-established procedures in the literature. Since the practical usefulness of a goodness-of-fit test is primarily determined by its ability to detect relevant alternatives with high probability in finite samples, a systematic numerical investigation is essential for assessing its effectiveness. All simulated data are generated as independent and identically distributed observations, consistent with the i.i.d. assumption underlying the proposed test.

To ensure a broad and meaningful assessment, we consider several parametric models that are widely used in reliability, survival analysis, actuarial science, and applied probability. These models represent different forms and intensities of positive aging and provide a natural benchmark for evaluating the sensitivity of the proposed procedure. Specifically, we consider the linear failure rate (LFR), Makeham, Gamma, and Weibull families. The LFR model generalizes the exponential distribution and is particularly useful for describing linearly increasing hazard behavior. The Makeham distribution is a flexible extension of the exponential law that allows more complex hazard structures and is frequently used in actuarial and demographic applications. The Gamma distribution is a standard model for waiting times and cumulative degradation phenomena, while the Weibull distribution remains one of the most important and versatile models in reliability analysis. For the Gamma and Weibull families, the IFRA property holds when the shape parameter exceeds one. The survival functions of the considered alternatives are summarized in Table 1.

**Table 1:** Survival functions of the alternative models considered in the simulation study.

Distribution	Survival Function	Support
LFR	${\bar{F}}_{1}(x)=e^{-\left(x+0.5\theta x^{2}\right)}$,	x>0,θ>0
Makeham	${\bar{F}}_{2}(x)=e^{-\left(x+\theta \left(x+e^{-x}-1\right)\right)}$,	x>0,θ>0
Gamma	${\bar{F}}_{3}(x)=\int_{x}^{\infty }\frac{t^{\theta -1}}{\Gamma (\theta )}e^{-t}dt\;$	x>0,θ>0
Weibull	${\bar{F}}_{4}(x)=e^{-x^{\theta }}$,	x>0,θ>0

To compute empirical rejection probabilities, we exploit the scale-invariant structure of the proposed test statistic. For each distribution and each selected parameter value θ, we generate independent samples of sizes *n* = 25, 50, and 100. The nominal significance level is fixed at α=0.05. For each sample size, the critical value of ${\hat{T}}^{*}$ is determined under the null hypothesis of exponentiality by means of 10,000 Monte Carlo replications. More precisely, we simulate 10,000 samples from the exponential model, compute the corresponding values of ${\hat{T}}^{*}$, and use the empirical (1−α)-quantile as the rejection threshold. For each alternative model, the empirical power is then estimated as the proportion of 10,000 independently generated samples for which the test statistic exceeds this critical value. This design provides a stable and fair numerical basis for comparing the proposed method with competing procedures.

To benchmark the performance of the proposed statistic, we compare it with a collection of established nonparametric tests for exponentiality against aging alternatives. These include the rank-based statistic *J*_0.5_ of Deshpande [4], the statistic *t*_1_(3,0.5) of Aly [17], the moment-based statistic $T_{n}$ of Jammalamadaka et al. [18], and the entropy-based procedures proposed by Alqefari [8], namely $\overset{*}{\underset{1,0.5;1^{1}}{\hat{\delta }}}\overset{*}{\underset{1,0.7;1^{1}}{,\;\hat{\delta }}},\;\overset{*}{\underset{1,0.8;1^{{\;}^{\prime }}}{\hat{\delta }}}\;\overset{*}{\underset{2,0.7;2}{\hat{\delta }}},\;\overset{*}{\underset{2,0.8;2^{{\;}^{\prime }}}{\hat{\delta }}}$ and $\overset{*}{\underset{4,0.5;2}{\hat{\delta }}}$. This collection represents several major methodological directions in the literature, including rank-based, moment-based, and information-theoretic approaches, and therefore provides a comprehensive basis for performance comparison. It is instructive to contrast the proposed statistic with existing IFRA tests. Deshpande s *J*_0.5_ [4] is a rank-based test that, while simple, does not directly measure average failure rate monotonicity. The moment-based test $T_{n}$ by Jammalamadaka et al. [18] exploits U-statistics but relies on asymptotic normality. The entropy-based tests by Alqefari [8] offer an information-theoretic perspective but lack exact finite-sample distributions. Our statistic ${\hat{𝐓}}^{⋆}$ differs in three fundamental ways: (i) it is constructed from the exact IFRA inequality, (ii) it admits an exact null distribution via exponential spacings, and (iii) it remains interpretable as an average aging measure.

To provide a complete assessment of finite-sample performance, the simulation study evaluates both empirical Type I error rates and empirical power. The rows corresponding to the exponential distribution in Tables 2–4 report the empirical sizes of the proposed test and the competing procedures under the null hypothesis at the nominal significance level (α=0.05). These empirical sizes were obtained using the same Monte Carlo design as in the power study, with samples generated from the exponential null distribution. Thus, they directly assess the ability of each procedure to preserve the prescribed significance level.

**Table 2:** Empirical Type I error rates and power comparisons of the tests at significance level 0.05 for *n* = 25.

Distribution	θ	$J_{0.5}$	$t_{1}(3,0.5)$	$T_{n}$	$\overset{1^{⋆}}{\underset{1,0.5}{\hat{\delta }}}$	$\overset{1^{⋆}}{\underset{1,0.7}{\hat{\delta }}}$	$\overset{1^{⋆}}{\underset{1,0.8}{\hat{\delta }}}$	$\overset{2^{⋆}}{\underset{2,0.7}{\hat{\delta }}}$	$\overset{2^{⋆}}{\underset{2,0.8}{\hat{\delta }}}$	$\overset{2^{⋆}}{\underset{4,0.5}{\hat{\delta }}}$	${\hat{T}}^{⋆}$
Exponential	1	0.0428	0.0524	0.0491	0.0512	0.0487	0.0518	0.0502	0.0552	0.0501	0.0486
	1.2	0.2921	0.2582	0.2903	0.3854	0.3449	0.3175	0.3883	0.3848	0.2948	0.3987
	1.5	0.3475	0.3034	0.3412	0.4378	0.3847	0.3714	0.4471	0.4474	0.3497	0.4321
LFR	2.0	0.4036	0.3565	0.3956	0.5174	0.4586	0.4419	0.5036	0.5042	0.4060	0.5345
	2.5	0.4541	0.3964	0.4470	0.5682	0.4963	0.4961	0.5603	0.5454	0.4662	0.5946
	3.0	0.4921	0.4317	0.4883	0.6161	0.5340	0.5295	0.5881	0.6099	0.4956	0.6111
	1.2	0.1947	0.1865	0.1946	0.2296	0.2044	0.2210	0.2131	0.2266	0.1988	0.2289
	1.5	0.2270	0.2185	0.2381	0.2677	0.2489	0.2499	0.2731	0.2541	0.2375	0.2674
Makeham	2.0	0.2758	0.2697	0.2896	0.3323	0.3063	0.3157	0.3033	0.3220	0.2977	0.3375
	2.5	0.3193	0.2977	0.3350	0.3820	0.3541	0.3622	0.3704	0.3807	0.3316	0.3910
	3.0	0.3596	0.3352	0.3720	0.4420	0.3860	0.3875	0.4240	0.4057	0.3734	0.4199
	1.2	0.1490	0.1327	0.1396	0.1397	0.1461	0.1385	0.1305	0.1372	0.1464	0.1308
	1.5	0.3681	0.3220	0.3688	0.3566	0.3554	0.3417	0.2948	0.3030	0.3466	0.3057
Gamma	2.0	0.7577	0.6627	0.7560	0.7236	0.7350	0.7565	0.6048	0.6414	0.7136	0.6346
	2.5	0.9344	0.8603	0.9390	0.9237	0.9333	0.9358	0.8434	0.8560	0.9253	0.8564
	3.0	0.9881	0.9526	0.9894	0.9799	0.9841	0.9857	0.9486	0.9501	0.9796	0.9572
	1.2	0.2399	0.2111	0.2430	0.2476	0.2579	0.2335	0.2262	0.2247	0.2270	0.2175
	1.5	0.7061	0.6026	0.7172	0.7326	0.7089	0.7268	0.6715	0.6844	0.6823	0.7053
Weibull	2.0	0.9868	0.9555	0.9919	0.9960	0.9920	0.9909	0.9897	0.9902	0.9833	0.9923
	2.5	0.9998	0.9985	0.9999	1.0000	1.0000	1.0000	0.9999	0.9998	1.0000	0.9999
	3.0	0.9999	1.0000	1.0000	1.0000	1.0000	1.0000	1.0000	1.0000	1.0000	1.0000

**Table 3:** Empirical Type I error rates and power comparisons of the tests at significance level 0.05 for *n* = 50.

Distribution	θ	$J_{0.5}$	$t_{1}(3,0.5)$	$T_{n}$	$\overset{1^{⋆}}{\underset{1,0.5}{\hat{\delta }}}$	$\overset{1^{⋆}}{\underset{1,0.7}{\hat{\delta }}}$	$\overset{1^{⋆}}{\underset{1,0.8}{\hat{\delta }}}$	$\overset{2^{⋆}}{\underset{2,0.7}{\hat{\delta }}}$	$\overset{2^{⋆}}{\underset{2,0.8}{\hat{\delta }}}$	$\overset{2^{⋆}}{\underset{4,0.5}{\hat{\delta }}}$	${\hat{T}}^{⋆}$
Exponential	1	0.0471	0.0544	0.0544	0.0474	0.0475	0.0543	0.048	0.0499	0.0487	0.0498
	1.2	0.5010	0.4376	0.5007	0.6673	0.5704	0.5594	0.6384	0.6384	0.4729	0.6832
	1.5	0.5664	0.5056	0.5620	0.7206	0.6582	0.6392	0.7190	0.6992	0.5548	0.7506
LFR	2.0	0.6571	0.5790	0.6565	0.8161	0.7458	0.7213	0.8069	0.7971	0.6435	0.8258
	2.5	0.7350	0.6486	0.7238	0.8682	0.8013	0.7883	0.8572	0.8474	0.6962	0.8608
	3.0	0.7601	0.6962	0.7700	0.9008	0.8472	0.8209	0.8846	0.8760	0.7574	0.9140
	1.2	0.3028	0.2902	0.3209	0.3780	0.3630	0.3436	0.3808	0.3728	0.3279	0.3684
	1.5	0.3781	0.3455	0.3933	0.4677	0.4438	0.4106	0.4533	0.4341	0.3810	0.4481
Makeham	2.0	0.4683	0.4242	0.4812	0.5852	0.5551	0.5253	0.5660	0.5618	0.4736	0.5488
	2.5	0.5402	0.4912	0.5581	0.6485	0.6139	0.6052	0.6293	0.6367	0.5613	0.6247
	3.0	0.5994	0.5576	0.6224	0.7295	0.6752	0.6674	0.6889	0.7083	0.6141	0.7084
	1.2	0.2099	0.1873	0.2338	0.1946	0.2147	0.2082	0.1731	0.1859	0.2039	0.1801
	1.5	0.6263	0.5159	0.6560	0.5914	0.6167	0.6072	0.5112	0.5007	0.5957	0.5005
Gamma	2.0	0.9679	0.9031	0.9767	0.9546	0.9645	0.9647	0.9020	0.9059	0.9511	0.8947
	2.5	0.9996	0.9918	0.9995	0.9982	0.9991	0.9993	0.9896	0.9914	0.9967	0.9936
	3.0	1.0000	0.9989	1.0000	1.0000	1.0000	1.0000	0.9991	0.9999	1.0000	0.9996
	1.2	0.3978	0.3234	0.4173	0.4200	0.4252	0.3995	0.3895	0.4002	0.3803	0.3833
	1.5	0.9367	0.8691	0.9579	0.9629	0.9548	0.9512	0.9424	0.9421	0.9245	0.9519
Weibull	2.0	1.0000	0.9988	1.0000	1.0000	1.0000	1.0000	1.0000	1.0000	1.0000	1.0000
	2.5	1.0000	1.0000	1.0000	1.0000	1.0000	1.0000	1.0000	1.0000	1.0000	1.0000
	3.0	1.0000	1.0000	1.0000	1.0000	1.0000	1.0000	1.0000	1.0000	1.0000	1.0000

**Table 4:** Empirical Type I error rates and power comparisons of the tests at significance level 0.05 for *n* = 100.

Distribution	θ	$J_{0.5}$	$t_{1}(3,0.5)$	$T_{n}$	$\overset{1^{⋆}}{\underset{1,0.5}{\hat{\delta }}}$	$\overset{1^{⋆}}{\underset{1,0.7}{\hat{\delta }}}$	$\overset{1^{⋆}}{\underset{1,0.8}{\hat{\delta }}}$	$\overset{2^{⋆}}{\underset{2,0.7}{\hat{\delta }}}$	$\overset{2^{⋆}}{\underset{2,0.8}{\hat{\delta }}}$	$\overset{2^{⋆}}{\underset{4,0.5}{\hat{\delta }}}$	${\hat{T}}^{⋆}$
Exponential	1	0.0508	0.0457	0.0479	0.0536	0.0532	0.0489	0.0484	0.0558	0.0579	0.0555
	1.2	0.7422	0.6810	0.7576	0.9199	0.8542	0.8447	0.9055	0.8884	0.7302	0.9281
	1.5	0.8246	0.7608	0.8248	0.9515	0.9108	0.8961	0.9467	0.9374	0.8028	0.9631
LFR	2.0	0.8891	0.8435	0.9054	0.9832	0.9624	0.9448	0.9758	0.9768	0.8758	0.9852
	2.5	0.9321	0.8909	0.9418	0.9901	0.9776	0.9691	0.9901	0.9852	0.9254	0.9940
	3.0	0.9564	0.9267	0.9603	0.9962	0.9892	0.9839	0.9938	0.9922	0.9425	0.9980
	1.2	0.5367	0.4489	0.5277	0.6427	0.5977	0.5751	0.6202	0.6132	0.5082	0.6193
	1.5	0.6249	0.5418	0.6160	0.7588	0.6986	0.6867	0.7259	0.7094	0.6103	0.7234
Makeham	2.0	0.7455	0.6513	0.7417	0.8530	0.8081	0.7997	0.8313	0.8274	0.7113	0.8362
	2.5	0.8344	0.7414	0.8178	0.9186	0.8914	0.8729	0.8920	0.8989	0.8064	0.9098
	3.0	0.8823	0.8012	0.8776	0.9456	0.9273	0.9167	0.9351	0.9296	0.8527	0.9417
	1.2	0.3444	0.2979	0.3612	0.3166	0.3347	0.3361	0.2965	0.2969	0.3220	0.2601
	1.5	0.8945	0.7949	0.9040	0.8559	0.8761	0.8784	0.7760	0.7921	0.8569	0.7619
Gamma	2.0	0.9998	0.9964	0.9997	0.9993	0.9996	0.9998	0.9948	0.9963	0.9992	0.9947
	2.5	1.0000	1.0000	1.0000	1.0000	1.0000	1.0000	1.0000	1.0000	1.0000	1.0000
	3.0	1.0000	1.0000	1.0000	1.0000	1.0000	1.0000	1.0000	1.0000	1.0000	1.0000
	1.2	0.6555	0.5605	0.6665	0.7033	0.6786	0.6620	0.6404	0.6210	0.5986	0.6559
	1.5	0.9988	0.9925	0.9994	0.9996	0.9991	0.9997	0.9989	0.9989	0.9970	0.9991
Weibull	2.0	1.0000	1.0000	1.0000	1.0000	1.0000	1.0000	1.0000	1.0000	1.0000	1.0000
	2.5	1.0000	1.0000	1.0000	1.0000	1.0000	1.0000	1.0000	1.0000	1.0000	1.0000
	3.0	1.0000	1.0000	1.0000	1.0000	1.0000	1.0000	1.0000	1.0000	1.0000	1.0000

The results show that the proposed test maintains empirical sizes close to the nominal level across all considered sample sizes. This provides clear evidence of satisfactory size control and supports the finite-sample calibration of the proposed procedure. Importantly, this behavior is consistent with the exact normalized-spacings representation established under exponentiality, which provides a rigorous basis for null calibration beyond purely asymptotic arguments. The remaining rows in Tables 2–4 report empirical power under the LFR, Makeham, Gamma, and Weibull alternatives. The results for *n* = 25, *n* = 50, and *n* = 100 are presented in Tables 2–4, respectively, offering a comprehensive comparison of all procedures across the selected models and parameter values.

Tables 2–4 reveal several clear and consistent patterns regarding the performance of the proposed test relative to competing procedures. First, the empirical power of all procedures increases with the sample size, confirming the consistency of the competing tests as well as that of the proposed statistic. More importantly, the proposed test ${\hat{T}}^{*}$ exhibits remarkably stable and competitive performance across a wide range of alternatives and sample sizes. In many scenarios, it attains the highest or near-highest empirical power, particularly for moderate and large sample sizes. This consistent behavior provides strong numerical evidence for the effectiveness of the proposed functional construction.

For the LFR alternatives, the proposed test demonstrates clear dominance. Even for the relatively small sample size *n* = 25, it consistently achieves the highest empirical power for several parameter values. This advantage becomes more pronounced as the sample size increases, where the proposed statistic uniformly outperforms all competing procedures. These results indicate that the test is highly sensitive to gradual departures from exponentiality induced by linearly increasing hazard rates. As seen in Table 2, even at the relatively small sample size $n=25,{\hat{T}}^{*}$ yields the largest power among all competing procedures for θ=1.2, 2.0, and 2.5, with values 0.3987, 0.5345, and 0.5946, respectively.

A similar pattern is observed in Table 3 for *n* = 50, where the proposed statistic again attains the highest power across all reported parameter values, for instance 0.6832 at θ=1.2 and 0.9140 at θ=3.0. The dominance becomes even more pronounced in Table 4 for *n* = 100, where ${\hat{T}}^{*}$ reaches 0.9281 at θ=1.2 and 0.9980 at θ=3.0, outperforming all competing procedures. These results indicate that the proposed test is highly sensitive to gradual departures from exponentiality induced by linear hazard growth.

For the Makeham alternatives, the proposed test remains highly competitive and frequently ranks among the best-performing procedures. Although the margin of superiority is less pronounced than in the LFR case, the proposed statistic consistently delivers strong power across all sample sizes and parameter values, demonstrating its robustness under more complex hazard structures. In Table 2, ${\hat{T}}^{*}$ achieves the largest empirical power at θ=2.0 and 2.5, and remains among the top procedures throughout the table. In Table 3, the proposed test is again highly competitive, with powers 0.5488, 0.6247, and 0.7084 at θ=2.0, 2.5, and 3.0, respectively. In Table 4, its performance remains excellent, reaching 0.8362, 0.9098, and 0.9417 for the same parameter values. These results show that the proposed statistic remains highly effective even when the underlying hazard structure departs from exponentiality in a subtle and nonlinear manner.

For the Gamma alternatives, the proposed test remains competitive, although it does not always dominate the competing methods. This behavior is expected, as Gamma distributions can induce aging patterns that are particularly favorable to certain classical and entropy-based tests. Nevertheless, the proposed statistic maintains strong discriminatory power and performs reliably, especially for moderate to large deviations from exponentiality. For example, in Table 2, the proposed test yields power 0.6346 at θ=2.0, which is lower than several competitors, although it still displays substantial discriminatory ability. Similarly, in Table 3, the power is 0.8947 at θ=2.0, and in Table 4 it is 0.9947, which remains very high in absolute terms even if not always maximal. This behavior is not surprising: the Gamma alternatives can induce aging patterns that are especially well captured by some classical and entropy-based procedures. Nevertheless, the proposed test retains strong power and remains practically reliable, particularly once the deviation from exponentiality becomes moderate or large.

For the Weibull alternatives, the proposed test again exhibits strong performance, achieving power values that are comparable to the best competing procedures. For moderate and large parameter values, all methods attain near-perfect power, reflecting the substantial separation between the null and alternative hypotheses. In this regime, the proposed test remains fully competitive and demonstrates excellent sensitivity. In Table 2, the power is 0.7053 at θ=1.5, increasing to 0.9923 at θ=2.0, and becoming essentially perfect for larger values of the parameter. A similar pattern is seen in Tables 3 and 4, where the proposed test reaches 0.9519 at θ=1.5 for *n* = 50, and 0.9991 at the same parameter value for *n* = 100. For θ≥2, all procedures exhibit power extremely close to one, reflecting the strong separation between the null and alternative in these cases. Thus, although the proposed statistic is not uniformly dominant for all Weibull settings, it remains highly effective and performs at a level fully comparable to the best existing methods.

Taken together, Tables 2-4 reveal three broad features of the proposed procedure. First, the test is particularly effective for the LFR and Makeham families, where it often outperforms all competitors. Second, for Gamma and Weibull alternatives, it remains strongly competitive and achieves very high power whenever the departure from exponentiality is moderate to strong. Third, its performance improves steadily with sample size, reflecting the sound asymptotic behavior established in the theoretical section. In particular, the strong results already observed at *n* = 25 suggest that the proposed method benefits substantially from its exact finite-sample foundation, which helps maintain reliable behavior even outside the purely asymptotic regime.

Although the proposed test demonstrates strong empirical performance, several limitations should be noted. First, the present formulation is developed for complete and uncensored lifetime data. Therefore, direct application to right-censored or truncated survival data would require suitable methodological extensions. Second, as with many tests for exponentiality against aging alternatives, the power of the proposed statistic may be more limited when the departure from exponentiality is very weak, particularly for small sample sizes or for alternatives close to the exponential boundary. Third, the exact finite-sample calibration, while providing an important advantage in terms of size control, may become computationally more demanding for very large samples; in such cases, the asymptotic approximation provides a practical alternative. Fourth, the method is developed under the classical assumption of independent and identically distributed continuous lifetimes. Applications involving dependent, discrete, heavily tied, or highly contaminated observations may require additional diagnostic checks or modified procedures. These limitations do not undermine the validity of the proposed framework, but they clarify its intended scope and identify natural directions for future research, including extensions to censored data, dependent lifetime sequences, and robust or resampling-based implementations.

## 5 Real data applications and empirical validation

To demonstrate the practical effectiveness and interpretability of the proposed statistic ${\hat{T}}^{*}$, we analyze several real-world datasets arising from engineering, biomedical, environmental, and geological contexts. These datasets reflect fundamentally different underlying mechanisms and provide a comprehensive empirical validation of the proposed methodology across diverse application domains. A crucial preliminary step in such analyses is to obtain empirical insight into the underlying aging behavior prior to formal hypothesis testing. To this end, we employ the Total Time on Test (TTT) plot, a well-established graphical diagnostic introduced by Barlow and Campo [19]. Although primarily associated with the IFR property, the concavity of the TTT curve also provides strong indicative evidence for the broader IFRA class.

Let $X_{\left(1\right)}\leq \cdots \leq X_{\left(n\right)}$ denote the ordered observations. Define


$$\begin{array}{c} D_{k}=(n-k+1)\left(X_{\left(k\right)}-X_{\left(k-1\right)}\right),\;k=1,\ldots ,n, \end{array}$$

with *X*_(0)_ = 0, and cumulative sums $S_{i}=\sum_{k=1}^{i}\;D_{k}$. The TTT plot consists of the points $\left(i/n,S_{i}/S_{n}\right)$. Under exponentiality, the curve follows approximately the diagonal, whereas a concave shape indicates positive aging behavior All computations and graphical analyses in this section were carried out in April 2026.

### 5.1 Dataset 1: Aircraft failure data

The first dataset, originally reported by Proschan [20], consists of *n* = 29 inter-failure times (in operating hours) of air-conditioning systems in Boeing 720 aircraft. These data come from a famous study of reliability in aviation systems, where understanding failure patterns is critical for maintenance scheduling and safety. The specific observations represent the intervals between successive failures for a single aircraft, and have been ordered from smallest to largest. The observed failure times are:

10, 14, 20, 23, 24, 25, 26, 29, 44, 44, 49, 56, 59, 60, 61, 62, 70, 76, 79, 84, 90, 101, 118, 130, 156, 186, 208, 208, 310

**Fig 1:**
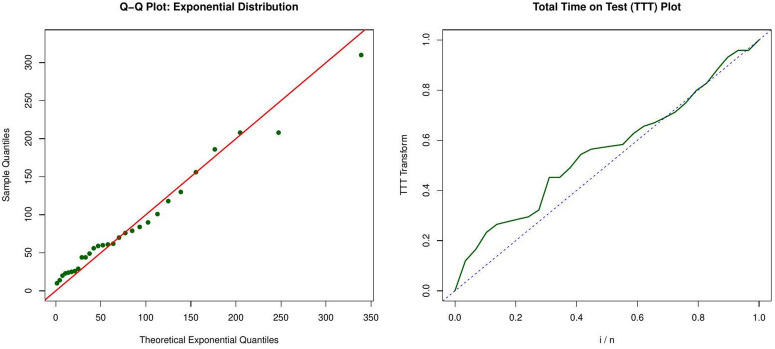
Exponential Q-Q plot and TTT plot for Dataset 1.

The graphical diagnostics in Fig 1 indicate that the TTT curve closely follows the reference diagonal, while the Q–Q plot exhibits near-linear alignment with the exponential model. These features suggest no substantial deviation from exponentiality. The formal test confirms this observation, yielding ${\hat{T}}^{*}=0.2244$ with a p-value exceeding 0.05. Therefore, the null hypothesis of exponentiality cannot be rejected. From a reliability perspective, this indicates that failures occur in a largely random manner, consistent with systems operating under stable conditions without pronounced aging effects.

### 5.2 Dataset 2: Leukemia survival data

The second dataset, obtained from Bryson and Siddiqui [21], contains *n* = 43 observations representing survival times (in days) of patients diagnosed with chronic granulocytic leukemia. These data are from a clinical study tracking patients from the time of diagnosis, providing insight into disease progression and patient prognosis. In survival analysis contexts, such data help identify whether the risk of death increases, decreases, or remains constant over time following diagnosis. The ordered survival times are:

7,47, 58, 74, 177, 232, 273, 285, 317, 429, 440, 445, 455, 468, 495, 497, 532, 571, 579, 581, 650, 702, 715, 779, 881, 900, 930, 968, 1077, 1109, 1314, 1334, 1367, 1534, 1712, 1784, 1877, 1886, 2045, 2056, 2260, 2429, 250

**Fig 2:**
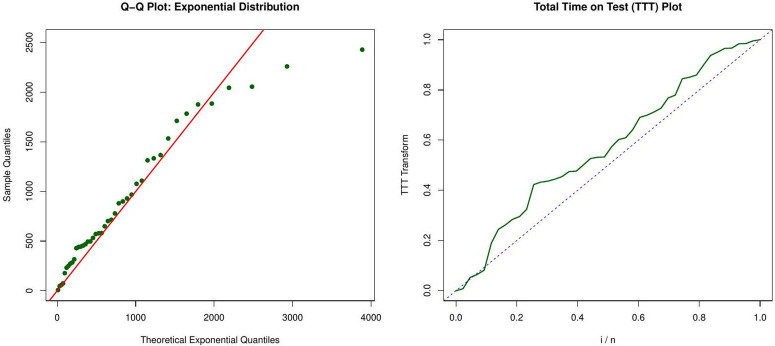
Exponential Q-Q plot and TTT plot for Dataset 2.

The graphical diagnostics in Fig 2 reveal a clearly concave TTT curve, indicating departure from exponentiality. The Q–Q plot further exhibits systematic curvature. The proposed test strongly supports this visual evidence, yielding a p-value below 0.05 and leading to rejection of the null hypothesis. This provides clear evidence of positive aging behavior, consistent with increasing hazard rates typically observed in disease progression. It is clear that rejection of exponentiality implies that patient mortality risk increases with time since diagnosis, supporting more frequent clinical monitoring as survival time lengthens.

### 5.3 Dataset 3: Flood data

The third dataset, reported by Seshadri [22], consists of *n* = 20 annual maximum flood discharges (in millions of cubic feet per second) of the Susquehanna River. In hydrology and environmental science, annual maximum flood series are standard for studying extreme events and assessing flood risks over time. A key question is whether flood risks increase due to cumulative environmental changes (such as land use modification or climate variability), making this dataset particularly relevant for testing aging behavior in a non-engineering context. The observed values are:

0.654, 0.613, 0.315, 0.449, 0.297, 0.402, 0.379, 0.423, 0.379, 0.3235, 0.269, 0.740, 0.418, 0.412, 0.494, 0.416, 0.338, 0.392, 0.484, 0.265

**Fig 3:**
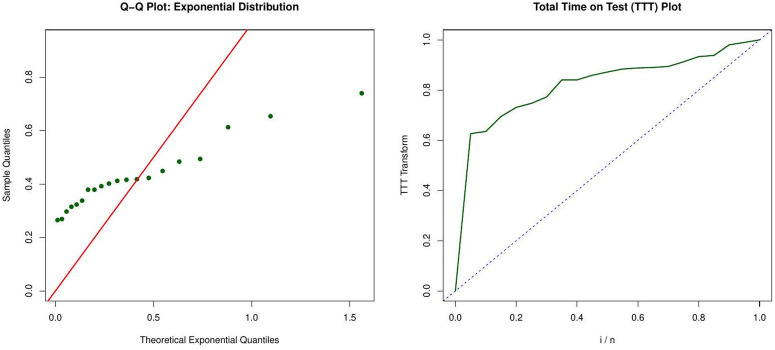
Exponential Q-Q plot and TTT plot for Dataset 3.

The TTT plot in Fig 3 exhibits pronounced concavity, providing strong evidence against exponentiality. The proposed test statistic exceeds the corresponding critical value, with a p-value well below 0.05, leading to a decisive rejection of the exponential model. This indicates IFRA-type behavior, suggesting that the likelihood of extreme flood events increases over time due to cumulative environmental effects.

### 5.4 Dataset 4: Aquifer thickness data

The fourth dataset, originally analyzed by Thomas and Jose [23], consists of *n* = 77 measurements of aquifer thickness obtained through geoelectrical resistivity methods. In hydrogeology, aquifer thickness is a critical parameter for groundwater resource assessment, as it influences water storage capacity and well yield. The data come from a groundwater exploration study in a crystalline basement complex, where geophysical techniques are used to identify potential zones for water development. In such contexts, progressive structural accumulation or geological processes may induce patterns analogous to IFRA aging. The observed values are:

10.49, 8.8, 12.42, 4.58, 6.85, 4.58, 5.0, 4.75, 4.75, 12.25, 9.5, 13.54, 10.42, 4.65, 9.88, 6.21, 8.6, 7.06, 7.96, 7.89, 9.7, 13.9, 12.65, 10.0, 12.65, 12.07, 9.8, 13.54, 9.82, 13.54, 12.42, 12.73, 12.22, 12.25, 12.32, 8.75, 12.0, 17.5, 11.88, 13.13, 13.56, 15.44, 13.22, 7.28, 11.7, 11.7, 11.6, 10.9, 11.84, 8.0, 10.2, 5.77, 13.9, 4.58, 12.07, 15.44, 10.2, 11.0, 8.5, 10.99, 10.39, 9.9, 13.94, 15.21, 13.56, 9.0, 20.47, 15.22, 11.5, 13.9, 13.22, 10.48, 15.48, 9.8, 12.21, 13.56, 7.04

**Fig 4:**
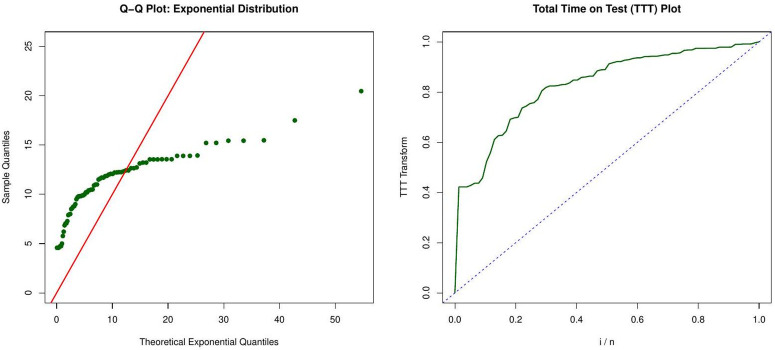
Exponential Q-Q plot and TTT plot for Dataset 4.

The graphical diagnostics in Fig 4 display strong concavity in the TTT plot, indicating a clear deviation from exponential behavior. The formal test confirms this finding, yielding a p-value far below 0.05. Although the notion of a failure rate is not directly applicable in a geological context, the observed statistical behavior aligns with IFRA-type patterns, suggesting progressive structural accumulation effects.

For practitioners, each of these dataset analyses yields a domain-specific interpretation. For the leukemia survival data (Dataset 2), rejection of exponentiality implies that patient mortality risk increases with time since diagnosis, suggesting that more frequent clinical monitoring is warranted as survival time lengthens. For the flood data (Dataset 3), the IFRA finding indicates that the likelihood of extreme flood events increases over time, which has implications for updating infrastructure design standards. For the aquifer thickness data (Dataset 4), although failure rate is not a geological concept, the statistical evidence of IFRA-type behavior suggests progressive structural accumulation effects that may inform groundwater resource assessment. In contrast, the aircraft failure data (Dataset 1) show no evidence of aging, supporting a constant-risk model for maintenance planning.

The combined use of graphical diagnostics and formal hypothesis testing provides a coherent and effective framework for assessing aging behavior in real-world systems. Across all datasets, the proposed statistic ${\hat{T}}^{*}$ shows strong agreement with empirical evidence, consistently distinguishing between exponential and IFRA-type behaviors. This stable performance across diverse application domains highlights the robustness, flexibility, and practical relevance of the proposed methodology, and further supports its applicability to real-world reliability and survival analysis problems.

Despite the structural interpretability and exact finite-sample advantages of the proposed nonparametric test, certain methodological limitations must be highlighted. First, the exact null representation and the normalized spacings framework derived in this study strictly rely on the assumption of i.i.d. complete lifetime data. In many practical clinical trials and engineering applications, data are frequently subject to censoring (such as right-censoring or interval-censoring). Applying the current test statistic directly to censored data without adjustments would induce bias, as the complete ordering and spacings would be disrupted. Second, the assumption of independence may not hold for systems exhibiting spatial clustering or shared environmental stresses, which introduce data dependencies. Finally, like many classical functional deviation tests, the proposed statistic lacks innate robustness against heavy data contamination or severe outliers, which can distort the calculated average failure rate curve. Adapting this testing framework to accommodate censored mechanisms, dependent structures, or contaminated datasets represents an important and necessary direction for further study.

## 6 Conclusion and future research

This paper has proposed an exact and interpretable functional test for exponentiality against IFRA alternatives, addressing a central inferential problem in reliability and survival analysis. The proposed statistic is derived directly from the defining structure of the IFRA class, thereby providing a transparent measure of departure from the no-aging exponential benchmark in terms of increasing average failure risk. A key feature of the method is its exact finite-sample representation under the exponential null hypothesis through normalized spacings. This representation provides a rigorous basis for finite-sample calibration and reduces dependence on purely asymptotic approximations. At the same time, the L-statistic structure of the proposed statistic yields asymptotic normality and supports efficient large-sample implementation. The scale-invariant formulation further enhances practical applicability by removing dependence on the unknown exponential parameter.

The simulation results demonstrate that the proposed test maintains satisfactory empirical size control and achieves competitive, and often superior, power compared with established procedures across a range of IFRA alternatives, including linear failure rate, Makeham, Gamma, and Weibull models. Its performance is particularly strong under LFR and Makeham alternatives, while remaining highly competitive for Gamma and Weibull models. The real-data applications further confirm the practical usefulness of the method, showing that it provides coherent and interpretable conclusions across diverse applied settings, including engineering reliability, biomedical survival analysis, environmental extremes, and geological measurements.

From an applied perspective, the proposed test offers a clear decision-support tool. In reliability engineering, rejection of exponentiality in favor of IFRA behavior indicates evidence of progressive deterioration, supporting age-based maintenance, inspection, or replacement policies that account for increasing failure risk over time. Failure to reject exponentiality suggests that a constant-risk model may be adequate, indicating that random shocks rather than cumulative deterioration may dominate the failure process. In biomedical survival analysis, rejection of exponentiality suggests that risk may increase with time after diagnosis or treatment, supporting the use of time-dependent prognostic models and potentially more intensive follow-up strategies. Thus, the proposed procedure is not only theoretically justified but also practically interpretable.

Several directions remain open for future research. First, the present framework is developed under the classical assumption of independent and identically distributed continuous lifetimes. This assumption is standard in nonparametric testing for exponentiality and is essential for the exact normalized-spacings representation derived in this paper. However, many reliability and survival datasets may exhibit temporal dependence, particularly in longitudinal, recurrent-event, or time-indexed monitoring settings. Extending the proposed framework to strictly stationary or weakly dependent lifetime sequences, possibly through mixing conditions, block-bootstrap calibration, or other resampling-based techniques, is an important direction for further study. Second, the functional construction developed here may be adapted to other aging classes, such as DMRL, NBU, and HNBUE. Third, bootstrap or permutation-based calibration may further improve finite-sample implementation when exact calibration becomes computationally demanding. Finally, extensions to right-censored, truncated, and multivariate lifetime data remain important open problems.

Moreover, the proposed test of this paper is developed for independent and identically distributed complete lifetime observations. Therefore, the present version is most appropriate when failure times are fully observed and may reasonably be treated as homogeneous and independent. Extensions to right-censored data, dependent lifetimes, contaminated samples, competing risks, or covariate-adjusted survival models require additional theoretical development and are important directions for future research.

Overall, the proposed method provides a unified, interpretable, and statistically rigorous framework for detecting IFRA-type aging in lifetime data. By combining a direct connection to the IFRA defining structure, exact finite-sample validity, asymptotic tractability, scale invariance, and strong empirical performance, the test offers a useful contribution to reliability analysis, survival modeling, and related applied fields.
